# Qualitative Research of Violent Incidents Toward Young Paramedics in the Czech Republic

**DOI:** 10.5811/westjem.2019.10.43919

**Published:** 2020-02-21

**Authors:** Jiří Knor, Jaroslav Pekara, Jana Šeblová, David Peřan, Patrik Cmorej, Jitka Němcová

**Affiliations:** * Emergency Medical Services of the Central Bohemian Region, Czech Republic; † Medical College in Prague, Prague, Czech Republic; ‡ Prague Emergency Medical Services, Czech Republic; § Charles University, 3rd Medical Faculty, Prague, Czech Republic; ¶ Emergency Medical Services of the Ústí nad Labem Region, Czech Republic; || Jan Evangelista Purkyně University, Division of Health Studies, Ústí nad Labem, Czech Republic

## Abstract

**Introduction:**

Prehospital and emergency medical services (EMS) providers are usually the first to respond to an individual’s urgent health needs, sometimes in emotionally charged circumstances. Because violence toward EMS providers in the Czech Republic is often overlooked and under-reported, we do not have a complete understanding of the extent of such violence, nor do we have recommendations from EMS professional organizations on how to resolve this problem in prehospital emergency medicine.

**Methods:**

We conducted this study to explore the process of violence against EMS providers, using the Strauss/Corbin systematic approach of grounded theory to create a paradigm model. The participants in this research included personnel who had at least two years experience in the EMS systems of the city of Prague and the Central Bohemian Region, and who had been victims of violence. Our sample included 10 registered paramedics and 10 emergency medical technicians ages 23–33 (mean ± standard deviation: 27.7). The impact of communication during EMS delivery, in the context of violence from patients or their relatives, emerged as the core category and the main focus of our study. The five main groups of the paradigm model of violence against EMS personnel included causal, contextual and intervening conditions, strategies, and consequences.

**Results:**

Of the 20 study participants, 18 reported experiencing an attack during the night shift. Ten participants experienced violence on the street, and 10 inside an ambulance. The perpetrators in all 18 cases were men. The behavior of EMS personnel plays a crucial role in how violent confrontations play out: nonprofessional behavior with drunken or addict patients increases the possibility of violence in 70% of cases.

**Conclusion:**

We found that paramedics and EMTs were exposed to verbal abuse and physical violence. However, in 10 of the violent encounters reported by our 20 participants, the attack was perpetrated by otherwise-ordinary people (ie, individuals with strong family support and good jobs) who found themselves in a very stressful situation. Thanks to grounded theory we learned that for all 20 participants there was a potential opportunity to prevent the conflict.

## INTRODUCTION

Violence toward prehospital emergency professionals is an often-neglected topic. There is no complete understanding of the incidence of violence in the Czech Republic, nor are there recommendations for specific professional communities regarding the problem of violence and how to resolve it in prehospital emergency care.^1^ Prehospital and emergency medical services (EMS) providers are the first to respond to medical emergencies. A high prevalence of violence has been reported in a few studies, indicating the extent of the problem. It also seems that one factor contributing to inappropriate patient behavior may be the nonprofessional conduct of some prehospital emergency personnel.^2^

The rate of occupational injuries among paramedics and other EMS professionals is eight times higher than the national average for all workers and twice as high as the rate for police officers. It seems that there is no occupational group with a higher injury or fatality rate than paramedics and EMS providers.^3^ The basic theories of violence include frustration, social learning, and a general pattern of violence, violence vs nonviolence, inequality, and subcultural and ecological theory. Theories of violence, including the state of “remaining marked for life,” “direct correlation between organizational effects and creating a safe environment,” EMS managers’ self-awareness, and other contributing factors toward moderating violence must also be taken into account.^4^ Although some safety measures are designed to reduce violence in emergency departments, few studies have focused on the prehospital emergency setting, with its unpredictable and unstructured environment.^5^ Studies that explain the process of violence are yet to be carried out. Quantitative research cannot properly explore the real causes/roots of violence against paramedics and EMS providers; thus, we believe a qualitative approach is needed to understand the phenomenon and provide a basis for the promotion of safety, health and efficiency among EMS personnel.^6^

## METHODS

Our main aim was to identify the impact of interpersonal communications in EMS delivery in the context of violence from patients or their relatives.

### Study design

We conducted this study to explore the process of violence in EMS using the Strauss/Corbin systematic approach of grounded theory to provide a paradigm model. Such methods are often followed when there is no definitive theory that defines a social phenomenon (such as violence). The participants in this research included EMS providers with at least two years of work experience in the EMS systems of Prague or the Central Bohemian Region, who had been victims of violence. Our sample included 10 registered paramedics and 10 emergency medical technicians (EMT) between the ages of 23–33 (mean ± standard deviation [SD]: 27.7). The educational level of the participants included 11 with high school diplomas (EMTs), nine with bachelor’s degrees (paramedics), and two with master’s degrees (paramedics). The providers became victims of violence after they were deployed to a scene to provide emergency care to traumatic or non-traumatic patients.

**Population Health Research Capsule**

What do we already know about this issue?

Violence toward emergency medical services (EMS) providers is an often-neglected topic.

What was the research question?

How does the behavior of EMS providers influence the occurrence of violent incidents with patients?

What was the major finding of the study?

While paramedics and emergency medical technicians were exposed to violence, we found that 50% of the time the acts were perpetrated by ordinary people under stress.

How does this improve population health?

Implementing training to improve the soft skills and communication styles of EMS staff would lessen violent encounters with patients in high-stress situations.

### Setting

**Figure 1:**
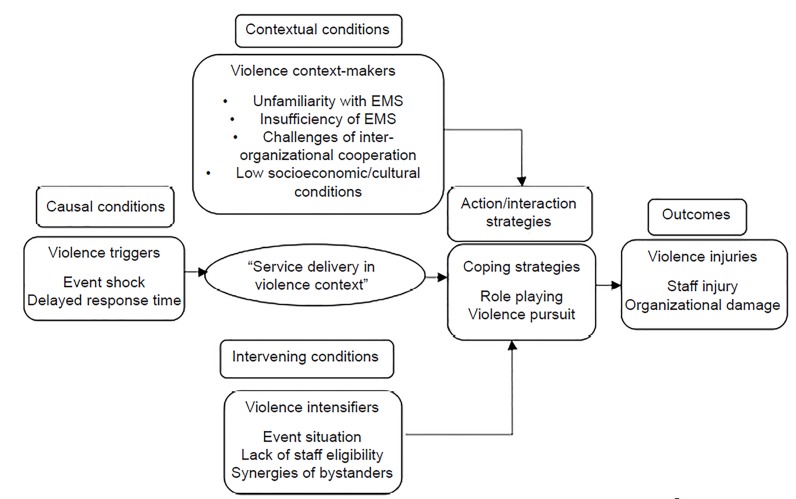
The paradigm model of workplace violence process in the emergency medical services setting.^7^ We encoded in each interview causal and contextual conditions, action/interaction strategies, and outcomes of every incident. *EMS*, emergency medical services.

The face-to-face interviews lasted from 20–50 minutes (mean±SD: 36.5) and were conducted in a location chosen by the participants. We collected data by means of a semi-structured interview, and all sessions were audio-recorded. We transcribed and analyzed the data using content analysis according to the Strauss/Corbin approach and constant comparative method to create a paradigm model of workplace violence (Figure 1). Our questions focused on the manner in which the violence occurred, how the EMS provider responded to the violence, and the consequences. In addition, we used observations and notes from documents and EMS medical records to document the following: circumstances of the event (eg, transporting the patient to the hospital); identification of the perpetrator’s role (eg, patient, family member of the patient, etc); whether the victim knew the perpetrator prior to the event; whether the respondent reported the assault to his or her employer; and any other conditions (eg, the perpetrator was intoxicated); and finally how the violence might have been averted.^7^

## RESULTS

In this study, the “impact of communication of emergency medical services delivery in the context of violence from patients or their relatives” emerged as the core category and the main focus. The five main groups of the paradigm model of violence against EMS staff included causal, contextual, and intervening conditions, strategies, and consequences (Figure 1).^7^

### Causal conditions

The main category of causal conditions was “triggers of violence,” which included two groups: “event shock” and “delayed response time.” Event shock refers to the prevalence of severe, unexpected events such as illness or trauma that may cause anxiety and agitation, resulting in unpredictable and uncontrollable behavior, such as violence: *“The father of the victim could no longer control himself and keep calm. His son had collapsed and he wanted to transport him quickly to the hospital. He verbally attacked the paramedics and couldn’t keep calm” (Participant 7)*

The second potential trigger of violence was delayed response time (RT). One of the major causes of violence in EMS conditions is the delay in RT, which can be due to a delay in requesting help, an imagined delay, unrealistic expectations, or actual delays in the arrival of EMS. Further delays can be due to staff negligence or a lack of resources, including the availability of an ambulance: *“People want us to be there immediately after the accident. That’s impossible. We arrived on the scene 10 minutes after the accident and the patient’s relative was waiting for us in front of their home. He was very angry, threatened us with his fists and was very rude, because we were late.” (Participant 5).*

### Contextual Conditions

This category, entitled “context-makers of violence,” includes four subgroups: unfamiliarity with EMS duties; insufficiencies of the EMS organization; challenges of inter-organizational cooperation; and disadvantaged socioeconomic and cultural conditions.

1. **Lack of familiarity.** The public’s unfamiliarity with EMS duties and inadequate knowledge of how the EMS system functions is illustrated by how the following request to transfer a non-emergency patient to the hospital resulted in violence: *“An 85-year-old man called an ambulance for his hypertension. I checked his blood pressure and it was OK (130/75). The patient showed us his homemade monitor for blood pressure control. The monitor showed normal parameters (120/80, 130/80, 125/70 – this was the last measurement). I tried to explain to him that his blood pressure is normal and everything is OK. He called for his wife and wanted his stick. Then he started banging his stick on the table and shouting: ‘You are only taxi drivers and I need to go to the hospital. Scoop me up and transfer me to the hospital!’”* (Participant 4)
2. **Insufficiencies of the EMS system**. One of the contextual factors of violence in the EMS resides in the insufficiencies of the EMS system itself, its regulations, and how providers are trained to manage situations. Communication between ambulance personnel and the relevant receiving centers can cause violence (overload of staff in the ambulance). *“We cannot take care of people and their things. We were looking after a 36-year-old man who got drunk and fell. He was verbally abusive, but at last he agreed to be transported to the hospital. In the courtyard of the hospital he started to stand up [inside the ambulance]and wanted to take off his seatbelt. I asked him to stay calm. He took a knife out of his jacket and cut the seatbelt and went toward me. At this moment my colleague (EMT) stopped the ambulance; I opened the door of the ambulance and locked the man inside. Then we called the police.”* (Participant 1)
3. **Challenge of inter-organizational cooperation**, and the performance of individuals from other organizations. The police are effective in ensuring the safety of EMS; however, in some scenarios we need the police to cooperate to calm the patient down*: “We wanted the help of the police, yes. But these policemen started to humiliate our patient and one of them wanted to hit him. We only wanted the police’s help for our safety during the transport to the hospital. The presence of police sometimes contributes to more violence from patients.”* (Participant 2)
4. **Poor socioeconomic and cultural conditions**. The incidence of violence is more prevalent in social environments with disadvantaged cultural, educational and socioeconomic status, and greater social malaise. Lack of interest on the part of the governmental health system in prevention of violence directed at EMS providers is another contributing factor. *“Most staff say – this is only part of the job, but I disagree. One 20-year-old man wanted to follow our ambulance in his car. I suspected he was drunk. I told him that if he followed us in his car I would call the police. Then he went over to me and hit me in the face.”* (Participant 17)

### Intervening conditions

Intervening conditions are a series of factors that impact violence strategies – time and place of the event, incompetence of EMS personnel, and involvement of bystanders.

1. **Time and place of the event**: Our goal was to determine when the prevalence of violence is the highest. Of the 20 respondents, 18 experienced the attack during the night shift (2–6 am); 10 experienced violence in the street, and 10 inside the ambulance.
2. **Incompetence of EMS personnel**: In some cases, due to conditions on the ground and the anxiety of clients, we see inappropriate behavior by EMS providers. This is a significant contributing factor in the occurrence of violence perpetrated against them. Thanks On the basis of grounded theory we found that all 20 participants had some chance to prevent their conflicts.
3. **Involvement of bystanders**: Some studies have pointed to high-risk groups in the context of violence against EMS personnel, such as people with a history of drug abuse, users of alcohol and psychedelic agents, and aggressive and irresponsible individuals with criminal records who are involved in the escalation of violence.^8^, ^9^ On the other hand, we found that in 10 cases among our 20 participants the attack was instigated by ordinary people (ie, those with stable families and good jobs) who were under extreme stress.

### Action/Interaction Strategies

In this study, we identified “coping strategies” as the main category with two groups: “role playing” and following up on violence.”

1. **Role playing**: EMS staff focused on providing optimal services by ignoring violence against them, exhibiting self-control, and managing violence through various strategies including explaining, convincing, relaxing, using confidence and self-defense techniques, such as leaving the scene, keeping away, and building trust, accepting the client’s demands, taking refuge, and seeking the cooperation of the perpetrator. Another strategy involves cooperation with the police, who play an important role at the scene to prevent violence or reduce injury: *“We heard the insults (‘We’re gonna kill you!’), but did not reply. We saw one man who tried to stand up and three people around him. We were still in the ambulance and the driver started to back up and then we went away and turned the corner. Then we called the police and cooperated with them”* (Participant 16)
2. **Following up on violence**: These strategies include reporting violence and protecting victims of violence. Especially in the case of EMS personnel who suffered physical injury, when they report violence to their supervisor they expect support, which depends on the sensitivity of the supervisor and the policy of their EMS organization. In some cases, violence is not reported for various reasons. The strategy of EMS management is to advise staff not to confront violence, and in the event of violence, to support the staff in their decision. Judicial support for victims of violence is another strategy used when following up on violence.

### Outcomes

Exposure to physical violence and verbal abuse puts EMS staff and organizations at risk of significant consequences.^10^

#### Staff injuries

Injuries to EMS personnel result in a variety of physical and psychological after-effects:

*“I am very careful now. One year after the incident (an unconscious man kicked me in the face when I tried to check him – I was knocked out for 10 minutes and out of duty for three months!); now when I am in contact with addicts during the night shift I remember it”* (Participant 20)

*“I wanted to call the police about a violent man and after I spoke he hit me in the face. He lives nearby our base. Whenever we go around there I remember this incident”* (Participant 9)

## DISCUSSION

In terms of “event shock” and “delayed response time,” paramedics often witness unpredictable and uncontrollable behavior such as violence. Delayed response time is one of the major causes of stress for EMS personnel, which can occur due to a client’s delay in requesting help, an imagined delay, unrealistic expectations, and actual delays in the arrival of EMS. Most studies on violence in healthcare have reported a close link between violence and stress. Once stress is intense and exceeds standard levels, it becomes a negative factor. A patient (but also a paramedic) who is unable to deal with stress can experience negative physical and mental responses (reduced self-control, unprofessional communication).^10^

Unfamiliarity with the role of EMS is another cause of conflicts, which can lead to disagreement with treatment interventions and refusal to accept services, and to the clients’ perception that EMS providers failed to meet their expectations; these were reported as the contextual factors of violence in some studies.^12^ On the other hand, we were witness to inappropriate communication from paramedics who seemed to devalue patients and their relatives.^13^ It would be useful to increase public awareness regarding the structure, capabilities and nature of prehospital emergency tasks, perhaps via the educational system and the media as a means of reducing the incidence of violence against EMS providers.

The insufficiency of the EMS organization itself is another contextual factor contributing to violence. Communication between ambulance staff and the relevant receiving centers can result in violence. In this section and in various studies, educational levels, competence, and the ability to assist clients, as well as a shortage of specialized staff, lack of experience and professional training, and low self-esteem were reported as underlying causes of violence.^14^ EMS managers must provide adequate and appropriate equipment/staff, create and maintain job satisfaction, and provide training on violence control as strategies to reduce workplace violence. In addition, measures must be taken to reduce violence and injury to health, while ensuring job satisfaction and providing equipment for the staff, teaching them self-defense, and cooperating with the police to protect EMS personnel as they provide service.^15^ The management of the EMS systems of Prague and the Central Bohemian Region (Czech Republic) provide for the use of personal protective gear, self-defense by means of evasion and pepper spray, training in how to keep a distance and how to transfer an aggressive client, use of restrictive agents, EMS managers also should emphasize the need for police involvement in cases of violence in order to establish security.

Other factors triggering violence ranged from the challenges of inter-organizational cooperation and disadvantaged socioeconomic and cultural conditions, including insulting, humiliating or irresponsive behavior, or even aggressiveness toward EMS personnel, to errors made by EMS providers themselves or negligence in duties, and fatigue caused by too many missions. In both sections, we found that EMS teams must cooperate with the police. Sometimes there are positive and negative aspects of this cooperation. One potential solution would be the creation of joint training and conferences (police + EMS).

Involvement of bystanders and high-risk groups, which include people with a history of drug abuse or who use alcohol and psychedelic agents, as well as people with a history of criminal and aggressive behavior, are central to the upsurge in violence against EMS personnel. The role that high-risk groups play in fomenting violence has been emphasized in similar studies. From our point of view, we believe it is necessary to create a database of scenarios and high-risk groups in cooperation with the police.^16^

Violence decreases job satisfaction, causes burnout, high staff turnover, and feelings of inadequate support, reduces the organization’s power, and ultimately impacts the performance and reputation of the EMS organization. Several related studies^17^, ^18^, ^19^ highlight the serious personal, organizational and professional consequences, as well as inadequate job safety, as some of the costs of violence.^20^, ^21^ Other related studies have mentioned minor and serious physical injury (eye and face injuries, bites, kicks, dislocations and fractures, bruises, and scratches) and psychological consequences such as stress irritability and headache, anxiety, depersonalization, depression, sleep disorders, irritability, fear of safety, and disturbing memories. Psychological injuries cause social consequences, including impact on social interactions, isolation, and personality changes in the workplace.^22^

## LIMITATIONS

Our study has several limitations. First, it was carried out at two institutions in the Czech Republic; thus, the results cannot be taken to be representative of all EMS systems within the Czech Republic as a whole or in other nations. Nevertheless, we have provided detailed information about the qualitative results of violence toward young paramedics and EMS personnel under specific conditions. A further limitation is that we focused only on young paramedics and EMTs, all of whom were men.

## CONCLUSION

Our study demonstrated that paramedics and EMTs were exposed to verbal abuse and physical violence. Of the 20 participants we interviewed, 18 reported being attacked during the night shift. Ten participants experienced violence in the street, and 10 inside the ambulance. In the 18 situations where EMS personnel encountered violence, all the perpetrators were men. We also found that the behavior of paramedics and EMTs plays a crucial role in escalating conflict. Specifically, nonprofessional behavior when confronted with drunk or drug-addicted patients increases the possibility of violence by 70%. On the other hand, we found that in 10 cases among our 20 participants the attack was caused by ordinary people under intense stress. Using the grounded theory approach we found that all 20 participants had some chance of preventing future conflicts from occurring.

## References

[1] Pekara J, Hulinsky P, Treslova M (2017). Prevalence of violence in nursing in the Czech Republic. J Nurs Care.

[2] Hahn S, Hantikainen V, Needham I (2012). Patient and visitor violence in the general hospital, occurrence, staff interventions and consequences: a cross-sectional survey. J Adv Nurs.

[3] Maguire BJ, Hunting KL, Guidotti TL (2005). Occupational injuries among emergency medical services personnel. Prehosp Emerg Care.

[4] di Martino V (2003). Workplace violence in the health sector. Relationship between stress and workplace violence in the health sector.

[5] Beech B, Leather P (2006). Workplace violence in the health care sector: a review of staff training and integration of training evaluation models. Aggress Violent Behav.

[6] Cresswell J (2012). Educational Research: Planning, Conducting, and Evaluating Quantitative and Qualitative Research.

[7] Pourshaikhian M, Abolghasem Gorji H, Aryankhesal A (2016). A systematic literature review: workplace violence against emergency medical services personnel. Arch Trauma Res.

[8] Gormley MA, Crowe RP, Bentley MA (2016). A national description of violence toward emergency medical services personnel. Prehosp Emerg Care.

[9] Gülen B, Serinken M, Hatipoğlu C (2016). Work-related injuries sustained by emergency medical technicians and paramedics in Turkey. Ulus Travma Acil Cerrahi Derg.

[10] Rahmani A, Hassankhani H, Mills J (2012). Exposure of Iranian emergency medical technicians to workplace violence: a cross-sectional analysis. Emerg Med Australas.

[11] Helge H, Cooper C (2000). Destructive conflict and bullying at work.

[12] Bernaldo-De-Quirós M, Piccini AT, Gómez MM (2015). Psychological consequences of aggression in pre-hospital emergency care: Cross sectional survey. Int J Nurs Stud.

[13] Hahn S, Müller M, Hantikainen K (2013). Risk factors associated with patient and visitor violence in general hospitals: Results of a multiple regression analysis. Int J Nurs Stud.

[14] Koritsas S, Coles J, Boyle M (2010). Workplace violence towards social workers: the Australian experience. Brit J Soc Work.

[15] Franz S, Zeh A, Schablon A (2010). Aggression and violence against health care workers in Germany: a cross sectional retrospective survey. BMC Health Serv Res.

[16] Piquero NL, Piquero AR, Craig JM (2013). Assessing research on workplace violence, 2000–2012. Aggress Violent Behav.

[17] Gates DM, Gillespie GL, Succop P (2011). Violence against nurses and its impact on stress and productivity. Nurs Econ.

[18] Grange JT, Corbett SW (2002). Violence against emergency medical services personnel. Prehosp Emerg Care.

[19] Bigham BL, Jensen JL, Tavares W (2014). Paramedic self-reported exposure to violence in the emergency medical services workplace: a mixed-methods cross-sectional survey. Prehosp Emerg Care.

[20] Furin M, Eliseo LJ, Langlois B (2015). Self-reported provider safety in an urban EMS. West J Emerg Med.

[21] Mechem D, Dickinson ET, Shofer FS (2002). Injuries from assaults on paramedics and firefighters in an urban emergency medical services system. Prehosp Emerg Care.

[22] Lanctôt N, Guay S (2014). The aftermath of workplace violence among healthcare workers: a systematic literature review of the consequences. Aggress Violent Beh.

